# Tapering and discontinuation of TNF-α blockers without disease relapse using ultrasonography as a tool to identify patients with rheumatoid arthritis in clinical and histological remission

**DOI:** 10.1186/s13075-016-0927-z

**Published:** 2016-02-03

**Authors:** Stefano Alivernini, Giusy Peluso, Anna Laura Fedele, Barbara Tolusso, Elisa Gremese, Gianfranco Ferraccioli

**Affiliations:** Institute of Rheumatology, Catholic University of the Sacred Heart, Rome, Italy; Division of Rheumatology, Institute of Rheumatology and Affine Sciences, Complesso Integrato Columbus, Via Giuseppe Moscati, 31, 00168 Rome, Italy

**Keywords:** Rheumatoid arthritis, Synovial tissue, Ultrasonography assessment, Disease remission, Anti-TNF-α agents, Discontinuation, Disease relapse

## Abstract

**Background:**

In this study, we assessed whether clinical and ultrasonography (US)-based remission could be used to select patients with rheumatoid arthritis (RA) eligible to taper and discontinue anti-TNF-α therapy after achievement of remission, looking at disease relapse.

**Methods:**

Forty-two patients with RA in sustained remission who were receiving anti-TNF-α treatment (Disease Activity Score <1.6 at three visits 3 months apart) underwent US evaluation of synovial hypertrophy (SH) and power Doppler (PD) signal presence. Five SH+/PD− patients with RA underwent US-guided knee synovial tissue biopsy to assess histological features of residual synovitis (CD68, CD3 and CD20 immunostaining) after sustained clinical remission was achieved. All patients were enrolled to taper first then discontinue anti-TNF-α. They were followed every 3 months afterwards, and the relapse rate was recorded.

**Results:**

Selected SH+/PD− patients showed low-grade synovitis as demonstrated by the presence of CD68+ cells in the lining layer and few infiltrating CD3+ and CD20+ cells at the time sustained clinical remission was achieved. After anti-TNF-α tapering, 13 patients (30.9 %) relapsed and 29 (69.1 %) SH+/PD− patients maintained disease remission after 3 months and discontinued anti-TNF-α treatment. Among them, 26 patients (89.7 %) maintained disease remission status after 6 months of follow-up. All patients who relapsed were retreated with the previous biologic, following the last effective therapeutic regimen, again reaching a good European League Against Rheumatism response within 3 months.

**Conclusions:**

US evaluation using PD signalling allows the identification of patients with RA in clinical and histological remission after tapering and discontinuing biologics.

**Electronic supplementary material:**

The online version of this article (doi:10.1186/s13075-016-0927-z) contains supplementary material, which is available to authorized users.

## Background

Persistent disease remission is the major goal of rheumatoid arthritis (RA) treatment. Current scoring methods based on composite indices cannot provide information on inflammation at the primary site of RA pathology, and joint damage progression can occur despite apparent clinical remission [1]. To date, no clear clinical parameters have been identified as being associated with disease flares [2]. Ultrasonography (US) has superior sensitivity in detecting the presence of synovial hypertrophy (SH) and its activity through power Doppler (PD) techniques in patients with RA [3, 4]. It has been demonstrated that patients with RA in clinical remission continue to have synovitis detectable through US and PD signalling, and that synovitis may be present in more than 50 % of subjects in remission on the basis of their Disease Activity Score in 28 joints (DAS28) [1]. We have previously demonstrated that US-PD remission occurs in half of patients with early RA and in a minority of patients with long-standing RA in clinical remission. Moreover, significantly fewer patients with RA with a negative PD signal detected by US evaluation had a flare during the 12-month follow-up period, compared with patients with RA who had a positive PD signal at the time remission was achieved [5]. Despite the known efficacy of anti-tumour necrosis factor (TNF)-α therapy for RA, cost [6] and safety issues [7] are among the concerns associated with prolonged use, which may lead physicians to consider discontinuation of anti-TNF-α treatment for patients with RA reaching sustained remission. Therefore, when, how and for whom to discontinue anti-TNF-α therapy are still unanswered questions in RA management.

On the basis of these concerns, we had four aims in the present study. First, we assessed the histological features of residual SH in patients with long-standing RA in clinical remission under combination therapy with methotrexate and anti-TNF-α agents. Second, we evaluated the percentage of patients who were selected on the basis of US and had successful discontinuation of therapy after biologic tapering. Third, we assessed whether US characteristics at the time of disease remission could help us to better discriminate final outcomes after anti-TNF-α therapy discontinuation. Fourth, we wanted to see whether reintroduction of the biologic could be successful in all patients.

## Methods

### Patient enrolment

Our monocentric, observational, prospective study cohort included 42 consecutive patients with long-standing RA (disease duration >12 months) in clinical remission (DAS <1.6 in three consecutive evaluations 3 months apart) who fulfilled the American College of Rheumatology (ACR) 2010 revised criteria for RA [8]. All patients with RA received combination therapy with methotrexate and TNF-α inhibitors (adalimumab 40 mg 2 weeks apart or etanercept 50 mg weekly). All patients with RA were taking methotrexate (mean dose 11.3 ± 2.6 mg/week). Demographic and clinical data were recorded for all the enrolled patients. Patients with RA in stable clinical remission (as previously assessed) were first tapered on anti-TNF-α therapy (adalimumab 40 mg/4 weeks or etanercept 50 mg/2 weeks) for 3 months. After 3 months from biologic tapering, patients who were still PD− discontinued anti-TNF-α therapy and were followed every 3 months afterwards while maintaining stable doses of methotrexate. The relapse rate was recorded for each patient (defined as change in DAS >1.2 from DAS value at time of US assessment) [9]. During the follow-up period, treatment modifications were not allowed. The study was approved by the ethics committee of Catholic University of the Sacred Heart, Rome, Italy, and informed consent was obtained from all patients before study entry.

### Laboratory assessment

Rheumatoid factor (RF) immunoglobulin A (IgA) and IgM (ORGENTEC Diagnostika, Mainz, Germany) and anti–cyclic citrullinated peptide (anti-CCP) autoantibodies (Axis Shield Diagnostics, Dundee, UK) were measured using a commercial enzyme-linked immunosorbent assay and performed according to the manufacturer’s instructions with the following proposed cut-off levels: 20 U/ml for IgA-RF and IgM-RF and 5 U/ml for anti-CCP.

### US assessment

All enrolled patients underwent US assessment according to the same protocol. Briefly, once clinical remission was achieved, each RA patient underwent US evaluation using greyscale and PD-US techniques in the following joint sites bilaterally: transverse and longitudinal scanning of dorsal and volar views of the second and third metacarpophalangeal (MCP) and proximal interphalangeal (PIP) joints and longitudinal and transverse scanning of the dorsal aspect of the wrist (radiocarpal–intercarpal), bilateral knee and second to fifth metatarsophalangeal (MTP) joints. We collected images of the ulnocarpal site (including assessment of tenosynovitis of the extensor carpi ulnaris tendon) to compare them with data from our previous study [5]. US assessment was performed by one rheumatologist (GP) experienced in US who was unaware of the clinical and laboratory findings. A commercially available real-time scanner (LOGIQ 9; GE Medical Systems, Milwaukee, WI, USA) equipped with a multi-frequency linear probe was used at 10–14 MHz. To reduce the possibility of bias, the patients were asked not to talk about their clinical symptoms with the US examiner. Each patient evaluation took nearly 30 minutes. Intra-reader reliability was evaluated by scoring 30 US images twice 1 month apart, and reliability was evaluated using the weighted κ statistics. Intra-reader reliability was 0.77.

The presence and location of any SH were quantified as thickness expressed in millimetres. SH was also graded on the basis of greyscale images using a semi-quantitative scoring method consisting of a 0–3 scale where 0 = no SH (defined as SH <2.0 mm for radiocarpal and intercarpal joints, SH <0.8 mm for second and third PIP joints and SH <0.5 mm for second and third MCP and second to fifth MTP joints), 1 = mild SH (defined as 2.0 mm < SH < 2.9 mm for radiocarpal and intercarpal joints, 0.8 mm < SH < 1.4 mm for second and third PIP joints and 0.5 mm < SH < 1.9 mm for second and third MCP and second to fifth MTP joints), 2 = moderate SH (defined as 3.0 mm < SH < 5 mm for radiocarpal and intercarpal joints, 1.5 mm < SH < 3.0 mm for second and third PIP joints and 2.0 mm < SH < 4.0 mm for second and third MCP and second to fifth MTP joints) and 3 = severe hypertrophy (defined as SH >5 mm for radiocarpal and intercarpal joints, SH >3.0 mm for second and third PIP joints and SH >4.0 mm for second and third MCP and second to fifth MTP joints). PD imaging was performed by selecting a region of interest that included the bone margins and the articular space. PD parameters were adjusted at the lowest permissible pulse repetition frequency to maximize sensitivity (900 Hz); low wall filters were used; Doppler frequency was 6.7 MHz; and the colour gain was set just below the level at which colour noise appeared under the bone surface. PD was recorded using a semi-quantitative technique consisting of a 0–3 scale where 0 = no PD signal, 1 = mild PD signal, 2 = moderate PD signal and 3 = marked PD signal. Two overall SH and PD scores were calculated as the sum of scores obtained from each joint for SH and PD [5].

### Synovial tissue biopsy of patients with RA in clinical remission and CD68, CD20 and CD3 immunostaining

At study entry, five SH+/PD− patients with RA (in clinical and US remission) provided informed consent and underwent knee synovial tissue (ST) biopsy according to the published standard procedure [10]. In this way, we assessed the histological features of residual SH of patients with RA in clinical remission (DAS <1.6). ST specimens were stained for cluster of differentiation 68 (CD68) mouse anti-human monoclonal antibody (clone 514H12), CD20 mouse anti-human monoclonal antibody (clone L26) or CD3 mouse anti-human monoclonal antibody (clone LN 10) (all from Leica Biosystems, Newcastle upon Tyne, UK) using Immunostainer BOND-MAX (Leica Biosystems).

### Statistical analysis

All statistical analyses were done using IBM SPSS 20.0 software (IBM, Armonk, NY, USA). Categorical variables were expressed as number and quantitative variables as mean ± standard deviation. Continuous data were analysed using parametric tests (independent *t* test), and ordinal data were analysed using a non-parametric Mann-Whitney *U* test. Categorical data were analysed using χ^2^ tests. Correlations were determined by Spearman’s rank order correlation. A *p* value <0.05 was considered statistically significant.

## Results

### Baseline demographic, immunological, US and histological characteristics of the RA cohort reaching DAS-based disease remission

**Fig. 1:**
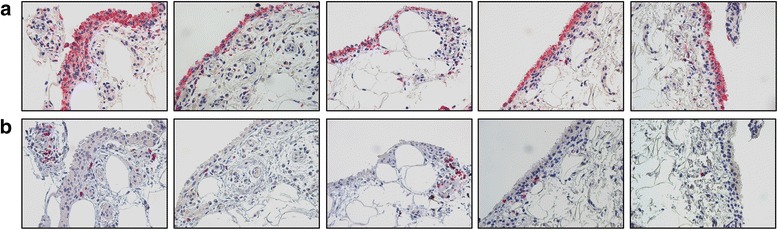
Cluster of differentiation 68 (CD68), CD20 and CD3 immunohistochemical staining of synovial tissue (ST) of patients with rheumatoid arthritis (RA) in clinical remission after undergoing therapy with tumour necrosis factor-α blockers. Five synovial hypertrophy–positive/power Doppler–negative patients with RA underwent ultrasonography-guided knee ST biopsy at study entry. **a** CD68 immunohistochemical staining of ST (original magnification, ×40). **b** CD20/CD3 double immunohistochemical staining of ST [CD20 diaminobenzidine (brown) and CD3 (red); original magnification, ×40]

Forty-two patients with RA [33 women (78.6 %)] who achieved persistent clinical DAS remission were enrolled in the study. Of note, using a more stringent definition such as Clinical Disease Activity Index (CDAI) remission, 15 patients with RA (35.7 %) in the general cohort were confirmed as being in clinical remission. The clinical and demographic characteristics of the patients are summarized in Additional file 1: Table S1. Five SH+/PD− patients with RA underwent ST biopsy at study entry. Immunostaining revealed very low-grade residual synovitis, as demonstrated by the presence of one to three layers of CD68+ cells (resident macrophages) in the lining and few CD3+ and CD20+ cells (T and B lymphocytes, respectively) (Fig. 1).

### Relapse rate after anti-TNF-α tapering in SH+/PD− patients with RA

**Fig. 2:**
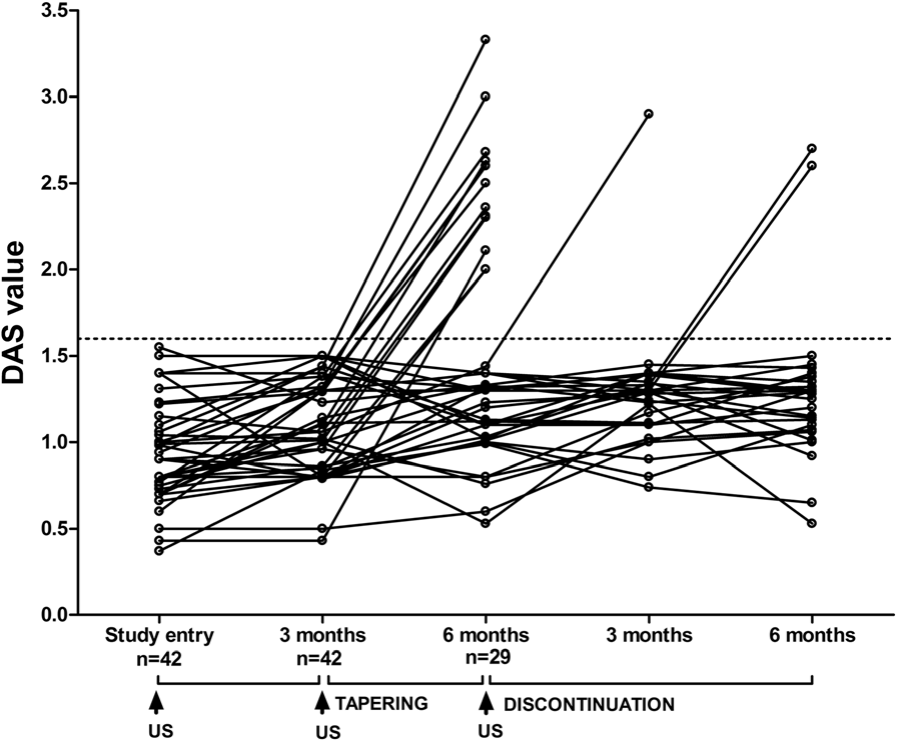
Changes in Disease Activity Score (DAS) values over time during the tapering and discontinuation of biologic treatment in synovial hypertrophy–positive (SH+)/power Doppler–negative (PD−) patients with rheumatoid arthritis (RA). Forty-two SH+/PD− patients with RA were tapered on anti-tumour necrosis factor (TNF)-α therapy for 3 months. Among them, 29 SH+/PD− patients with RA (69.1 %) maintained disease remission 3 months after anti-TNF-α tapering. All SH+/PD− patients still in disease remission after anti-TNF-α tapering discontinued anti-TNF-α treatment and were followed every 3 months afterwards. Among them, 26 (89.7 %) maintained disease remission for 6 months, whereas 3 (10.3 %) had disease relapse within 6 months. Among patients with RA who completed the 12-month follow-up, 16 SH+/PD− patients (38.1 %) had a chance of disease relapse after anti-TNF-α tapering and discontinuation. *US* ultrasonography

After 3 months from tapering, 13 patients with RA (30.9 %) had disease relapse (Fig. 2). Patients with RA who relapsed were not different from patients with RA who did not relapse with regard to anti-CCP (*p* = 0.89), IgA-RF (*p* = 0.86) or IgM-RF (*p* = 0.86) positivity; smoking habit (*p* = 0.34); or biologic type (53.8 % adalimumab-treated patients vs. 46.2 % etanercept-treated patients had disease relapse; *p* = 0.79). However, SH values were significantly higher at the second MCP and fifth MTP joints in the relapse group compared with the patients who did not relapse after 3 months on the lower-dose anti-TNF regimen (Table 1).

**Table 1:** Characteristics of SH+/PD− patients with RA who relapsed or did not after tapering or discontinuation of anti-TNF-α therapy

RA cohort^a^ (*n* = 42)	Relapse in tapering cohort (*n* = 42)			Relapse in discontinuation cohort (*n* = 29)		
	No (*n* = 29)	Yes (*n* = 13)	*P* value^b^	No (*n* = 26)	Yes (*n* = 3)	*P* value^c^
Age, yr	53.4 ± 11.4	53.7 ± 10.0	0.89	53.1 ± 11.0	53.4 ± 10.1	0.87
Disease duration, yr	9.8 ± 7.0	10.1 ± 6.9	0.75	9.8 ± 6.8	10.1 ± 6.7	0.86
Anti-TNF-α duration, yr	4.9 ± 1.9	5.0 ± 2.0	0.81	4.8 ± 1.7	5.2 ± 1.8	0.91
Females	23 (79.3)	10 (76.9)	0.86	20 (76.9)	3 (100.0)	0.35
Smoking	9 (31.0)	6 (46.2)	0.34	2 (7.6)	1 (33.3)	0.17
Baseline anti-CCP+	18 (62.1)	9 (69.2)	0.89	12 (46.2)	2 (66.7)	0.50
Baseline IgM-RF+	12 (41.4)	5 (38.5)	0.86	10 (38.5)	2 (66.7)	0.35
Baseline IgA-RF+	12 (41.4)	5 (38.5)	0.86	8 (30.8)	2 (66.7)	0.22
Low-dose prednisone <5 mg/day	3 (10.3)	3 (23.1)	0.28	2 (7.6)	0 (0.0)	0.62
Etanercept use	14 (48.3)	6 (46.2)	0.87	11 (42.3)	1 (33.3)	0.77
Adalimumab use	15 (51.7)	7 (53.8)	0.64	12 (46.2)	2 (66.7)	0.50
US parameters^d^						
Second MCP joint SH (V), mm	0.5 ± 0.2	0.6 ± 0.1	0.25	0.6 ± 0.2	0.7 ± 0.1	0.39
Second MCP joint SH (D), mm	0.4 ± 0.2	0.6 ± 0.1	**0.05**	0.5 ± 0.1	0.6 ± 0.1	0.53
Third MCP joint SH (V), mm	0.5 ± 0.2	0.6 ± 0.2	0.91	0.7 ± 0.3	0.7 ± 0.5	0.78
Third MCP joint SH (D), mm	0.5 ± 0.2	0.6 ± 0.4	0.77	0.5 ± 0.2	0.5 ± 0.3	0.38
Second PIP joint SH (V), mm	0.5 ± 0.2	0.4 ± 0.3	0.52	0.5 ± 0.2	0.6 ± 0.2	0.26
Second PIP joint SH (D), mm	0.7 ± 0.6	0.5 ± 0.5	0.48	0.5 ± 0.4	0.6 ± 0.2	0.52
Third PIP joint SH (V), mm	0.7 ± 0.3	0.5 ± 0.3	0.77	0.6 ± 0.4	0.5 ± 0.5	0.48
Third PIP joint SH (D), mm	0.5 ± 0.1	0.4 ± 0.2	0.65	0.5 ± 0.1	0.5 ± 0.3	0.23
Intercarpal SH, mm	2.0 ± 0.4	1.8 ± 0.8	0.59	1.9 ± 0.7	1.9 ± 0.9	0.38
Radiocarpal SH, mm	0.5 ± 0.3	0.4 ± 0.2	0.36	0.5 ± 0.2	0.5 ± 0.2	0.84
Knee SH, mm	2.6 ± 4.8	2.7 ± 4.7	0.59	2.6 ± 4.1	2.7 ± 4.3	0.81
Second MTP joint SH, mm	1.2 ± 0.9	1.1 ± 0.7	0.16	1.3 ± 0.7	1.2 ± 0.9	0.41
Fifth MTP joint SH, mm	0.4 ± 0.2	0.7 ± 0.4	**0.01**	0.4 ± 0.3	0.7 ± 0.4	**0.04**
Total SH score	1.1 ± 1.7	1.2 ± 1.6	0.76	1.1 ± 1.6	1.2 ± 1.7	0.59

*RA* rheumatoid arthritis, *TNF* tumour necrosis factor, *US* ultrasonography, *CRP* C-reactive protein, *CCP* cyclic citrullinated peptide, *Ig* immunoglobulin, *RF* rheumatoid factor, *MCP* metacarpophalangeal joint, *PIP* proximal interphalangeal joint, *MTP* metatarsophalangeal joint, *SH* synovial hypertrophy, *D* dorsal view, *V* volar view

Data are presented as mean ± standard deviation or count (%). The values refer to both sides as a mean. Boldface type indicates *p* < 0.05.

^a^Patients with RA with Disease Activity Score <1.6 in three consecutive evaluations 3 months apart

^b^Relapsed vs. no relapsed patients after anti-TNF-α tapering

^c^Relapsed vs. no relapsed patients after anti-TNF-α discontinuation

^d^US assessment done on the same day of treatment modification

### Relapse rate after anti-TNF-α discontinuation in SH+/PD− patients with RA

Patients with RA who were still SH+/PD− after tapering discontinued anti-TNF-α therapy. After 6 months from anti-TNF-α discontinuation, 26 patients (89.7 %) maintained disease remission and 3 (10.3 %) had disease relapse (one patient at 3 months and two patients at 6 months, respectively) (Fig. 2). All patients who relapsed had a flare in the joint clinically involved at disease onset (66.7 % in MCP joints and 33.3 % in knee joints, respectively). Patients with RA who relapsed did not differ with respect to demographic and immunologic parameters or biologic type (66.7 % adalimumab-treated patients vs. 33.3 % etanercept-treated patients had disease flare; *p* = 0.41). However, higher SH scores at the fifth MTP joint were noted in the relapse group (Table 1). On the basis of CDAI remission criteria, despite PD negativity, 2 (13.3 %) of 15 patients with RA experienced disease relapse after tapering TNF blocker treatment and 1 (7.7 %) of 13 patients with RA had disease relapse after biologic discontinuation, compared with 30.9 % and 10.3 % of patients with RA in DAS remission after biologic tapering (*p* = 0.18) and discontinuation (*p* = 0.79), respectively.

All patients with RA who relapsed were retreated with the previous anti-TNF-α following the last effective therapeutic regimen and within 3 months again had a good response as defined by European League Against Rheumatism (EULAR) criteria. None of the patients with RA who underwent ST biopsy experienced disease relapse after anti-TNF-α tapering or discontinuation within the 6-month follow-up period.

## Discussion

In a 6-month follow-up prospective study, we found that US evaluation at the time of clinical remission could be an important tool to select patients with RA in clinical and histological remission eligible to undergo biologic therapy tapering and discontinuation after US-based selection, without experiencing disease relapse.

To date, treatment strategies for RA have been aimed at reaching disease remission and preventing further joint damage. However, the definition of remission has been based exclusively on clinical indexes. Although the availability of composite scores allows definition of clinical remission based on DAS44 or DAS28, it has been demonstrated that progression of joint damage can occur despite DAS-based remission achievement [2]. Moreover, it is known that most patients with RA in clinical remission continue to have synovitis detectable by US [4]. Recently, PD-US evaluation was noted to have additional value in daily clinical practice to establish true disease remission [11]. Moreover, it is known that US-detected residual synovitis is frequent and predicts the risk of relapse and structural progression in patients with RA in clinical remission [11].

The presence of SH seems to be a frequent finding in patients with long-standing RA with DAS-based remission status, owing to the contribution of significant long-standing disease to synovial membrane hypertrophy. Recently, Anandarajah et al. demonstrated, in a limited cohort of patients with RA in clinical remission according to the ACR criteria after various treatments (anti-TNF-α therapy or conventional disease-modifying anti-rheumatic drugs (DMARDs)], that imaging and histological assessment documented a persistently active disease state mainly in patients with RA receiving conventional DMARD treatment [12]. In our study, we included a subgroup of patients with RA who reached sustained remission with combined methotrexate and anti-TNF-α treatment, showing that the absence of US activity was associated with almost normal findings at the synovial level in terms of CD68-, CD3- and CD20-positive cell infiltration [13, 14].

Moreover, an important aim in daily practice is the management of patients with RA with long-term biologic treatment in terms of long-standing safety and efficacy. To date, there is no clear evidence that helps in the selection of patients with RA in clinical remission who will maintain their remission status over time and then will be eligible first to taper and then to interrupt biologic treatment. Naredo et al. recently suggested that the presence of PD-detected synovitis is an independent predictor of biologic therapy tapering failure in a heterogeneous cohort of patients with RA in sustained clinical remission using different biologic agents [15]. We previously found that PD positivity was higher in patients with RA in DAS-based clinical remission who experienced short-term disease flares [5].

The daily management of patients receiving long-term biologic treatment is still a matter of debate. It is not clear how to select patients with RA in remission who are eligible for interruption of biologic treatment. The majority of the published studies are long-term extension clinical trials powered to test efficacy and not the success of anti-TNF-α therapy discontinuation once remission is achieved. To date, the PRESERVE trial [16] is the only study in which researchers have compared the effect of etanercept continuation (50 mg or 25 mg weekly) and discontinuation in patients with long-standing RA. The trial showed that 43 % of patients maintained remission or low disease activity after discontinuation.

In our study, 69.1 % of patients who tapered anti-TNF-α therapy using US selection criteria maintained remission within 12 weeks afterwards, suggesting that there is a meaningful, large patient population with established RA in remission for whom anti-TNF-α dose can be decreased without clinical and functional worsening [16]. The US selection criteria seem to be a more realistic parameter to consider in patients with long-standing RA who are less likely to fulfil the CDAI remission criteria, mainly because of the patients’ reported CDAI item values.

Moreover, no clear data are available about the wisest strategy for tapering biologics without increasing the relapse rate. In our study, despite limitations linked to the single evaluating sonographer, we combined serial US evaluation with DAS as an additive decisional tool to select SH+/PD− patients eligible for tapering and discontinuation of anti-TNF-α therapy. Through this strategy 69.1 % of patients with RA maintained remission after anti-TNF-α tapering. Among patients with RA who successfully tapered anti-TNF-α treatment, 89.7 % maintained disease remission at 6-month follow-up after anti-TNF-α discontinuation. In addition, disease flares after anti-TNF-α discontinuation occurred in the joints with higher SH scores and clinically involved at disease onset, despite the fact that no SH cutoff discriminated patients who relapsed from those who did not. This finding suggests the possible utility of following US with indices of joints [17] initially involved at disease onset with higher likelihood of relapse.

This study has some limitations. It must be taken into account that increased SH score was associated with disease relapse only at certain joint sites. In particular, the fifth MTP joint was informative (in both the tapering and discontinuation groups) and the second MCP joint was informative for the tapering group only. US evaluation of all other assessed joints seemed not to predict relapse. Clearly, the fifth MTP joint is an important site for erosion [18], but the difference in SH measurements between the groups was small at 0.3 mm. While inter-reader reliability of US SH measurements was could not be assessed in our study, Ikeda and co-workers investigated this at the second MCP joint and found an intra-class correlation coefficient of 0.63, suggesting only moderate agreement between readers [19]. Finally, all the patients who relapsed after anti-TNF-α discontinuation reached a good EULAR response once anti-TNF-α therapy was reinitiated following the last effective scheme. This finding suggests that if an attempt to discontinue anti-TNF-α fails, a return to the previous disease control therapy is possible.

## Conclusions

Our study shows that US is a useful tool in the routine assessment of patients with long-standing RA who have achieved clinical remission while on combination therapy. US evaluation of SH and PD can be used to identify those patients in real clinical and histological remission. Moreover, the combination of PD-US evaluation and ACR/EULAR remission criteria could help identify patients on biologics who are likely to achieve drug-free remission. In these patients, withdrawal of therapy may be possible. Finally, the use of three sequential US evaluations may allow identification of an even higher proportion of those likely to reach persistent drug-free remission compared with using current clinical methods of disease activity assessment [20]. Confirmation of these findings is required in other patient cohorts.

## Additional file

Additional file 1: Table S1. Demographic, immunological, and US characteristics of patients with RA included in the observational study cohort. (DOC 51 kb)

## Abbreviations

ACR: American College of Rheumatology
CCP: cyclic citrullinated peptide
CD: cluster of differentiation
CDAI: Clinical Disease Activity Index
D: dorsal view
DAS: Disease Activity Score
DMARD: disease-modifying anti-rheumatic drug
EULAR: European League Against Rheumatism
Ig: immunoglobulin
MCP: metacarpophalangeal
MTP: metatarsophalangeal
PD: power Doppler
PIP: proximal interphalangeal
RA: rheumatoid arthritis
RF: rheumatoid factor
SD: standard deviation
SH: synovial hypertrophy
ST: synovial tissue
TNF: tumour necrosis factor
US: ultrasonography
V: volar view

## References

[1] Yoshimi R, Hama M, Takase K, Ihata A, Kishimoto D, Terauchi K (2012). Ultrasonography is a potent tool for the prediction of progressive joint destruction during clinical remission of rheumatoid arthritis. Mod Rheum.

[2] Molenaar ET, Voskuyl AE, Dinant HJ, Bezemer PD, Boers M, Dijkmans BA (2004). Progression of radiologic damage in patients with rheumatoid arthritis in clinical remission. Arthritis Rheum.

[3] Cheung PP, Dougados M, Gossec L (2010). Reliability of ultrasonography to detect synovitis in rheumatoid arthritis: a systematic literature review of 35 studies (1,415 patients). Arthritis Care Res (Hoboken).

[4] Brown AK, Quinn MA, Karim Z, Conaghan PG, Peterfy CG, Hensor E (2006). Presence of significant synovitis in rheumatoid arthritis patients with disease-modifying antirheumatic drug induced clinical remission: evidence from an imaging study may explain structural progression. Arthritis Rheum.

[5] Peluso G, Michelutti A, Bosello SL, Gremese E, Tolusso B, Ferraccioli G (2011). Clinical and ultrasonographic remission determines different chances of relapse in early and long standing rheumatoid arthritis. Ann Rheum Dis.

[6] Ollendorf DA, Klingman D, Hazard E, Ray S (2009). Differences in annual medication costs and rates of dosage increase between tumor necrosis factor-antagonist therapies for rheumatoid arthritis in a managed care population. Clin Ther.

[7] Bongartz T, Sutton AJ, Sweeting MJ, Buchan I, Matteson EL, Montori V (2006). Anti-TNF antibody therapy in rheumatoid arthritis and the risk of serious infections and malignancies: systematic review and meta-analysis of rare harmful effects in randomized controlled trials. JAMA.

[8] Aletaha D, Neogi T, Silman AJ, Funovits J, Felson DT, Bingham CO (2010). Rheumatoid arthritis classification criteria: an American College of Rheumatology/European League Against Rheumatism collaborative initiative. Arthritis Rheum.

[9] Alten R, Pohl C, Choy EH, Christensen R, Furst DE, Hewlett SE (2011). Developing a construct to evaluate flares in rheumatoid arthritis: a conceptual report of the OMERACT RA Flare Definition Working Group. J Rheumatol.

[10] Humby F, Kelly S, Bugatti S, Manzo A, Filer A, Mahto A (2016). Evaluation of minimally invasive, ultrasound-guided synovial biopsy techniques by the OMERACT filter-determining validation requirements. J Rheumatol.

[11] Nguyen H, Ruyssen-Witrand A, Gandjbakhch F, Constantin A, Foltz V, Cantagrel A (2014). Prevalence of ultrasound-detected residual synovitis and risk of relapse and structural progression in rheumatoid arthritis patients in clinical remission: a systematic review and meta-analysis. Rheumatology (Oxford).

[12] Anandarajah A, Thiele R, Giampoli E, Monu J, Seo GS, Feng C (2014). Patients with rheumatoid arthritis in clinical remission manifest persistent joint inflammation on histology and imaging studies. J Rheumatol.

[13] Singh JA, Arayssi T, Duray P, Schumacher HR (2004). Immunohistochemistry of normal human knee synovium: a quantitative study. Ann Rheum Dis.

[14] Della Beffa C, Slansky E, Pommerenke C, Klawonn F, Li J, Dai L (2013). The relative composition of the inflammatory infiltrate as an additional tool for synovial tissue classification. PLoS One.

[15] Naredo E, Valor L, De la Torre I, Montoro M, Bello N, Martínez-Barrio J (2015). Predictive value of Doppler ultrasound-detected synovitis in relation to failed tapering of biologic therapy in patients with rheumatoid arthritis. Rheumatology (Oxford).

[16] Smolen JS, Nash P, Durez P, Hall S, Ilivanova E, Irazoque-Palazuelos F (2013). Maintenance, reduction, or withdrawal of etanercept after treatment with etanercept and methotrexate in patients with moderate rheumatoid arthritis (PRESERVE): a randomised controlled trial. Lancet.

[17] Naredo E, Valor L, De la Torre I, Martínez-Barrio J, Hinojosa M, Aramburu F (2013). Ultrasound joint inflammation in rheumatoid arthritis in clinical remission: how many and which joints should be assessed?. Arthritis Care Res.

[18] Hulsmans HM, Jacobs JW, van der Heijde DM, van Albada-Kuipers GA, Schenk Y, Bijlsma JW (2000). The course of radiologic damage during the first six years of rheumatoid arthritis. Arthritis Rheum.

[19] Ikeda K, Seto Y, Narita A, Kawakami A, Kawahito Y, Ito H (2014). Ultrasound assessment of synovial pathologic features in rheumatoid arthritis using comprehensive multiplane images of the second metacarpophalangeal joint: identification of the components that are reliable and influential on the global assessment of the whole joint. Arthritis Rheumatol.

[20] Navarro-Millán I, Sattui SE, Curtis JR (2013). Systematic review of tumor necrosis factor inhibitor discontinuation studies in rheumatoid arthritis. Clin Ther.

