# Supplementary material for: Tapering and discontinuation of TNF-α blockers without disease relapse using ultrasonography as a tool to identify patients with rheumatoid arthritis in clinical and histological remission
Source: Arthritis Res Ther. 2016 Feb 3;18:39. doi: 10.1186/s13075-016-0927-z (PMC4741059; doi:10.1186/s13075-016-0927-z)
Supplement: Additional file 1: Table S1. — Demographic, immunological, and US characteristics of patients with RA included in the observational study cohort. (DOC 51 kb) [file 13075_2016_927_MOESM1_ESM.doc]

**Additional file 1: Table S1. Demographic, immunological and US characteristics of RA patients included in the observational study cohort.**

|  | **RA**  **Cohort***  **(n=42)** |
| --- | --- |
| **Age, yrs mean SD** | 54.0 ± 13.4 |
| **Disease duration, yrs mean SD** | 10.9 ± 7.2 |
| **Biologic tp duration, yrs mean SD** | 4.1 ± 2.2 |
| **Female, n (%)** | 33 (78.6) |
| **Smoking, n (%)** | 15 (35.7) |
| **Anti-CCP+, n (%)** | 27 (64.3) |
| **IgM-RF+, n (%)** | 17 (40.5) |
| **IgA-RF+, n (%)** | 17 (40.5) |
| **SH+PD- status, n (%)** | 42 (100) |
| **US parameters**** |  |
| **II MCP SH (V), mm, mean ± SD** | 0.6 ± 0.2 |
| **II MCP SH (D), mm, mean ± SD** | 0.5 ± 0.2 |
| **III MCP SH (V), mm, mean ± SD** | 0.6 ± 0.1 |
| **III MCP SH (D), mm, mean ± SD** | 0.6 ± 0.3 |
| **II PIP SH (V), mm, mean ± SD** | 0.5 ± 0.3 |
| **II PIP SH (D), mm, mean ± SD** | 0.6 ± 0.5 |
| **III PIP SH (V), mm, mean ± SD** | 0.6 ± 0.4 |
| **III PIP SH (D), mm, mean ± SD** | 0.5 ± 0.3 |
| **Intercarpal SH, mm, mean ± SD** | 2.1 ± 1.4 |
| **Radiocarpal SH, mm, mean ± SD** | 0.5 ± 0.2 |
| **Knee SH, mm, mean ± SD** | 2.7 ± 4.9 |
| **II MTP SH, mm, mean ± SD** | 1.1 ± 0.9 |
| **V MTP SH, mm, mean ± SD** | 0.5 ± 0.3 |
| **Total SH score, mean ± SD** | 1.2 ± 1.7 |

**RA**: Rheumatoid Arthritis patients; *RA patients with DAS<1.6 in 3 consecutive evaluations 3 months apart; ** US assessment was done on the same day of treatment modification; **anti-CCP**: anti-cyclic citrullinated peptides antibodies; **RF**:Rheumatoid Factor; **SD**: Standard deviation; **MCP:** Metacarpophalangeal joint; **PIP:** Proximal interphalangeal joint; **MTF:** Metatarsalphalageal joint**; SH:** synovial hyperthrophy; **(D):** Dorsal view; **(V)** Volar view; **mm:** millimeter; The values refer to both sides as a mean;
